# Supplementary figures and images for: A New Paramoeba Isolate From Florida Exhibits a Microtubule‐Bound Endosymbiont Closely Associated With the Host Nucleus
Source: J Eukaryot Microbiol. 2025 May 15;72(3):e70011. doi: 10.1111/jeu.70011 (PMC12079164; doi:10.1111/jeu.70011)

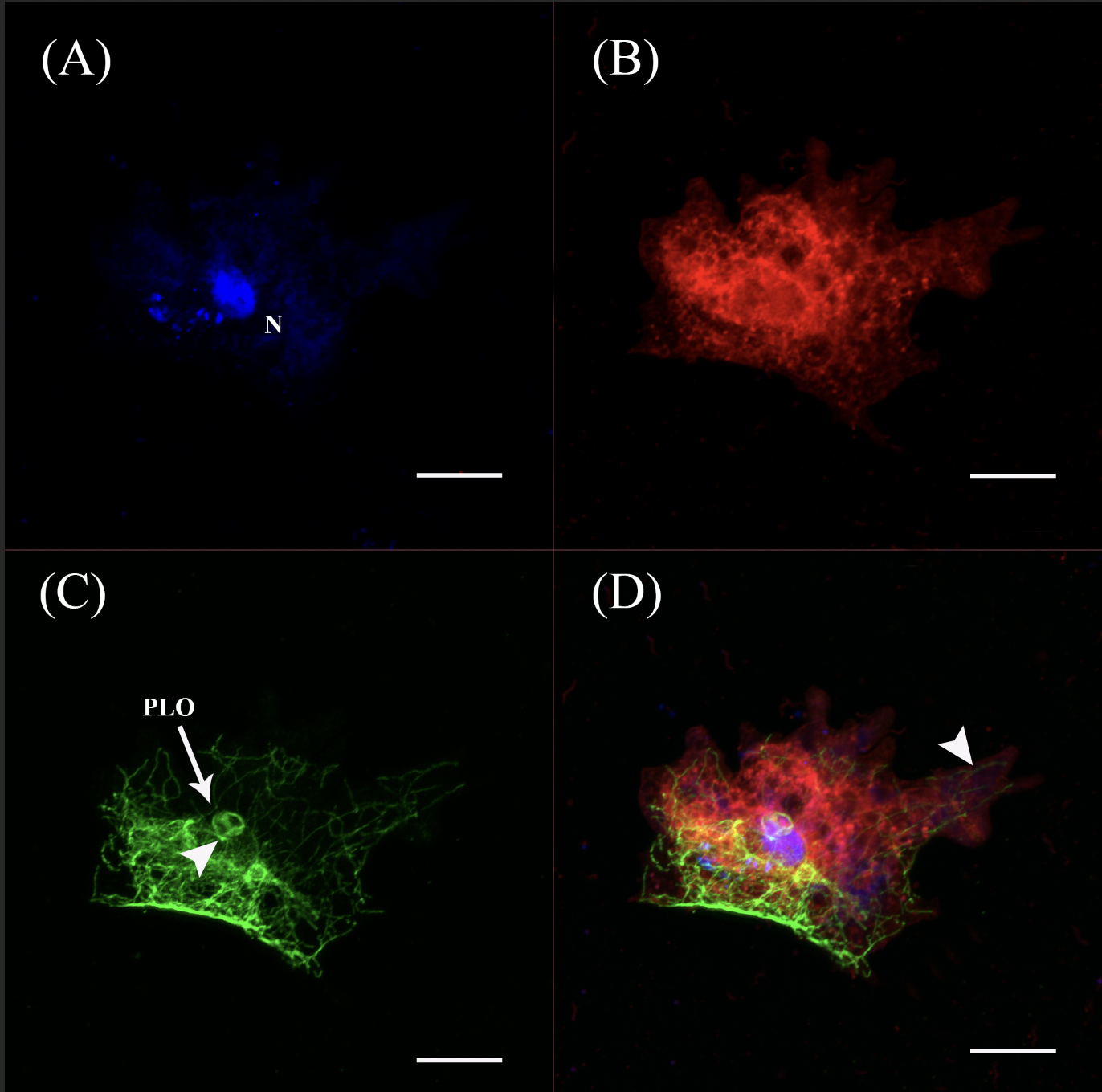

Supplement: Supplementary file 2 — Figure S1. Confocal maximum intensity projection split images of triple staining in Paramoeba daytoni n. sp., showing DNA (blue, A), microtubules (green, B), plasma membrane (red, C), and a merged image of all three (D). The staining reveals a well‐preserved network of host amoeba microtubules (MTs) along with two PLO‐associated MTs (C, D). Notably, the host MTs surrounding the nucleus form a network that appears to support the host nucleus, with a weak connection observed between these MTs and the PLO MTs (arrowhead, C). The subpseudopodia are supported by a few extending MTs (arrow, D). [file JEU-72-e70011-s008.png]

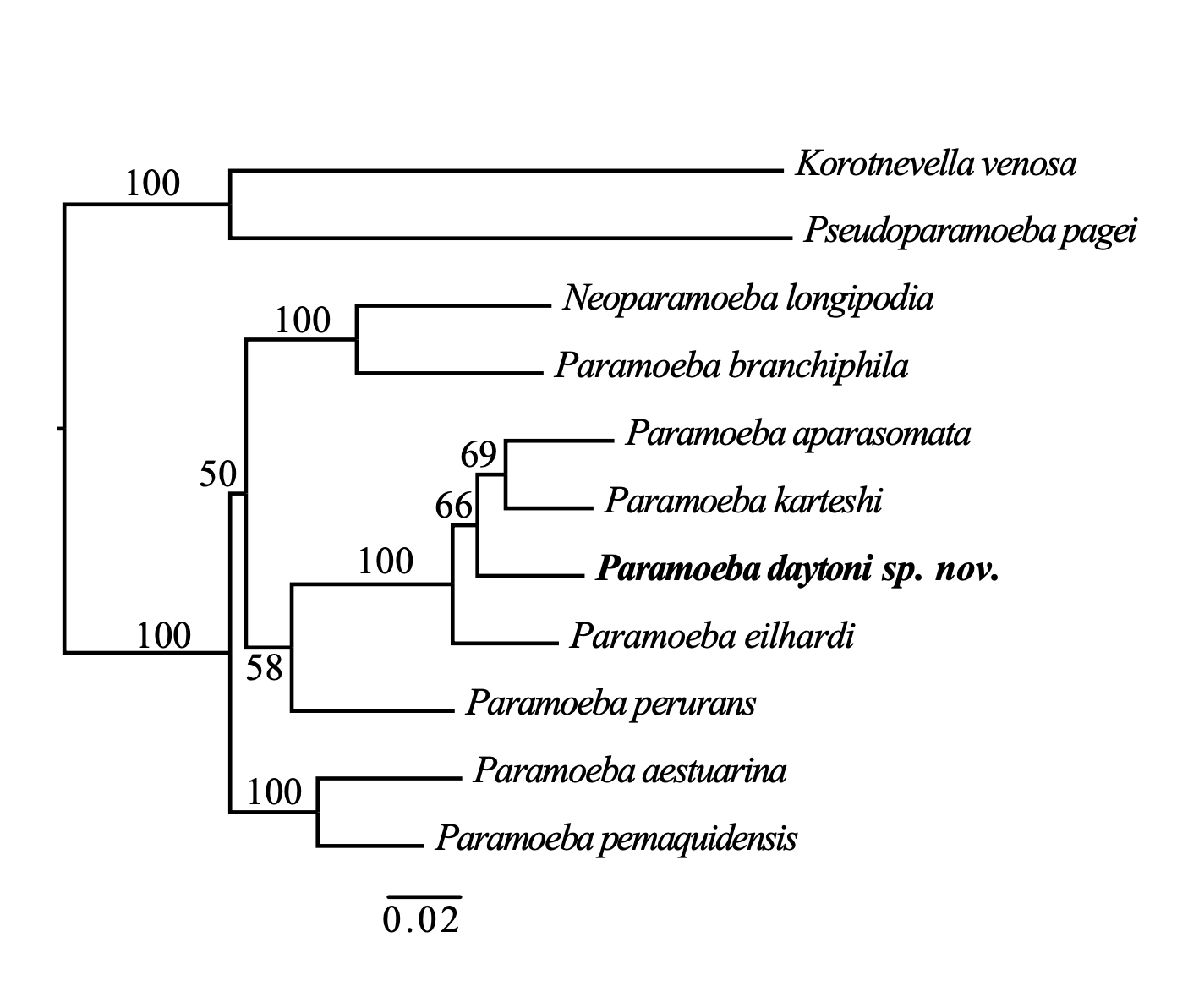

Supplement: Supplementary file 3 — Figure S2. IQ‐TREE maximum likelihood phylogeny based on nine ingroup taxa combining 18S and COI genes of Paramoeba/Neoparamoeba clade species, highlighting the position of Paramoeba daytoni n. sp. The alignment, generated using AliView, includes 2699 nucleotide positions. Numbers at nodes represent IQ‐TREE bootstrap values. Branches are drawn to scale. [file JEU-72-e70011-s001.png]

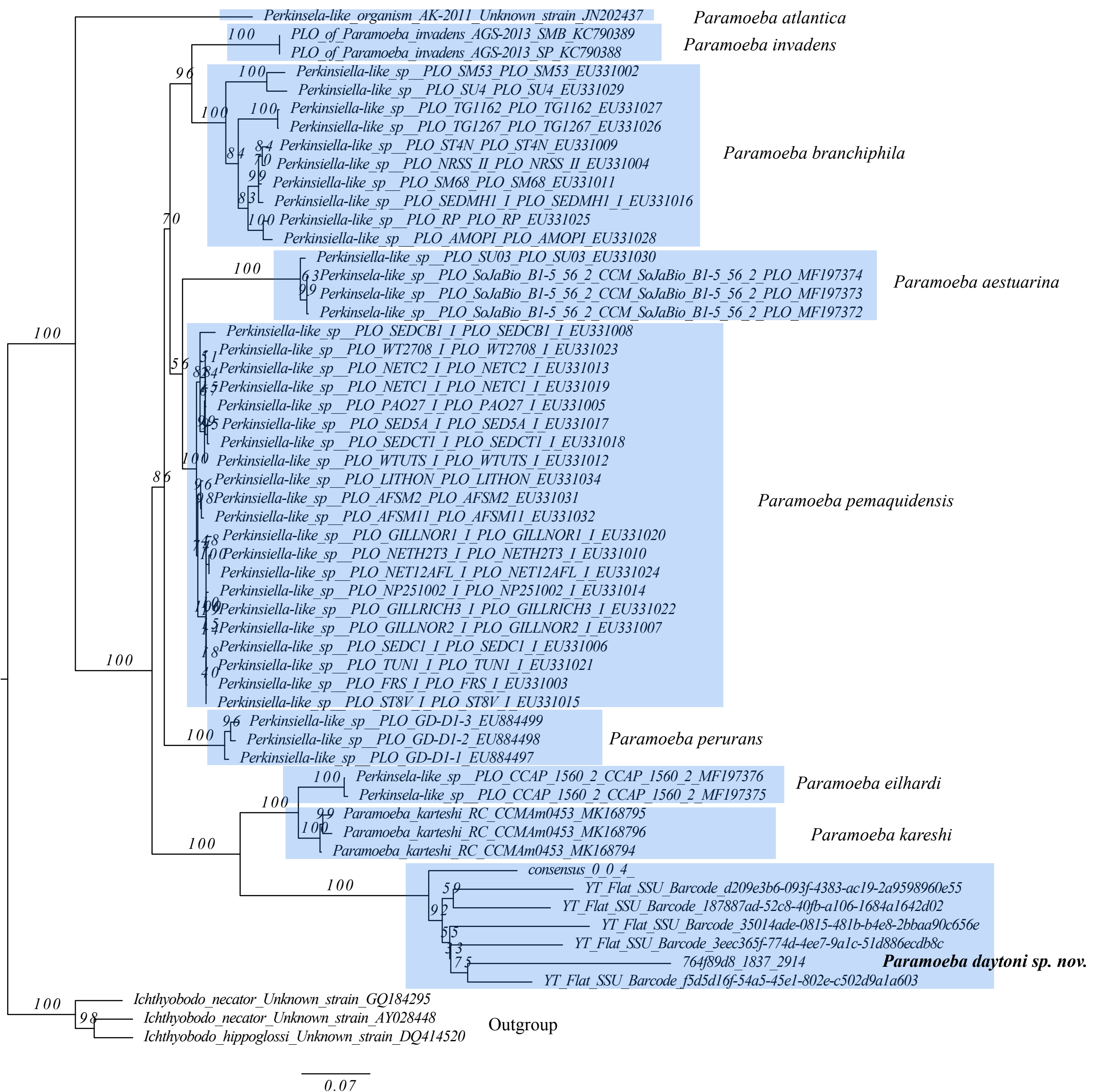

Supplement: Supplementary file 4 — Figure S3. IQ‐TREE maximum likelihood phylogeny based on PLO 18S gene of Paramoeba/Neoparamoeba clade species, highlighting the position of the PLO of Paramoeba daytoni n. sp. Numbers at nodes represent IQ‐TREE bootstrap values. Branches are drawn to scale. [file JEU-72-e70011-s002.pdf]
