# Supplementary material for: A New Paramoeba Isolate From Florida Exhibits a Microtubule‐Bound Endosymbiont Closely Associated With the Host Nucleus
Source: J Eukaryot Microbiol. 2025 May 15;72(3):e70011. doi: 10.1111/jeu.70011 (PMC12079164; doi:10.1111/jeu.70011)
Supplement: Supplementary file 5 — Table S1. SSU‐rDNA (18S) Intrastrain Variation of Paramoeba/Neoparamoeba species. [file JEU-72-e70011-s003.docx]

**Table S1.** SSU-rDNA (18S) Intrastrain Variation of *Paramoeba/ Neoparamoeba* species.

| **Species** | **Minimum** | **Maximum** | **Average** |
| --- | --- | --- | --- |
| *Paramoeba invadens* | 0.08% | 2.68% | 1.16% |
| *Paramoeba branchiphila* | 0.80% | 4.97% | 2.61% |
| *Neoparamoeba longipodia* | 0.05% | 1.33% | 0.98% |
| *Paramoeba perurans* | 0.00% | 1.67% | 0.81% |
| *Paramoeba eilhardi* | 1.76% | 2.54% | 2.12% |
| *Paramoeba karteshi* | 1.21% | 1.64% | 1.38% |
| *Paramoeba daytoni* | 0.00% | 1.71% | 0.94% |
| *Paramoeba aparasomata* | 0.49% | 2.31% | 1.79% |
| *Paramoeba pemaquidensis* | 0.00% | 3.58% | 2.03% |
| *Paramoeba aestuarina* | 0.14% | 5.54% | 3.26% |
