# Supplementary material for: A New Paramoeba Isolate From Florida Exhibits a Microtubule‐Bound Endosymbiont Closely Associated With the Host Nucleus
Source: J Eukaryot Microbiol. 2025 May 15;72(3):e70011. doi: 10.1111/jeu.70011 (PMC12079164; doi:10.1111/jeu.70011)
Supplement: Supplementary file 6 — Table S2. SSU‐rDNA (18S) interspecific variation of Paramoeba/Neoparamoeba clades. [file JEU-72-e70011-s007.docx]

**Table S2.** SSU-rDNA (18S) interspecific variation of *Paramoeba/ Neoparamoeba* clades.

| **Species Pair** | **Minimum** | **Maximum** | **Average** |
| --- | --- | --- | --- |
| *Neoparamoeba longipodia vs Paramoeba invadens* | 6.41% | 8.90% | 7.80% |
| *Paramoeba branchiphila vs Paramoeba invadens* | 6.23% | 9.96% | 7.62% |
| *Paramoeba branchiphila vs Neoparamoeba longipodia* | 6.43% | 9.30% | 7.77% |
| *Paramoeba karteshi vs Paramoeba eilhardi* | 3.54% | 4.16% | 3.92% |
| *Paramoeba daytoni vs Paramoeba eilhardi* | 3.27% | 4.64% | 3.99% |
| *Paramoeba daytoni vs Paramoeba karteshi* | 2.70% | 3.70% | 3.19% |
| *Paramoeba aparasomata vs Paramoeba eilhardi* | 3.74% | 5.14% | 4.29% |
| *Paramoeba aparasomata vs Paramoeba karteshi* | 2.67% | 3.23% | 2.89% |
| *Paramoeba aparasomata vs Paramoeba daytoni* | 2.25% | 3.75% | 2.99% |
| *Paramoeba aestuarina vs Paramoeba pemaquidensis* | 3.84% | 6.69% | 5.21% |
