# Supplementary material for: A New Paramoeba Isolate From Florida Exhibits a Microtubule‐Bound Endosymbiont Closely Associated With the Host Nucleus
Source: J Eukaryot Microbiol. 2025 May 15;72(3):e70011. doi: 10.1111/jeu.70011 (PMC12079164; doi:10.1111/jeu.70011)
Supplement: Supplementary file 7 — Table S3. Clade support and recovery across datasets aligned and analyzed using different phylogenetic algorithms. [file JEU-72-e70011-s006.docx]

**Table S3. Clade Support and Recovery Across Datasets Aligned and Analyzed Using Different Phylogenetic Algorithms.**

Taxon and clade name key: ***IBL*** *- (P. invadens + P. branchiphila) + Neoparamoeba longipodia);* ***PA*** *- P. pemaquidensis + P. aestuarina;* ***Pe*** *- P. perurans;* ***E*** *- P. eilhardi;* ***D*** *- P. Dayton sp. n.;* ***A*** *- P. aparasomata;* ***K*** *- P. kareshi.*
