# Supplementary material for: A New Paramoeba Isolate From Florida Exhibits a Microtubule‐Bound Endosymbiont Closely Associated With the Host Nucleus
Source: J Eukaryot Microbiol. 2025 May 15;72(3):e70011. doi: 10.1111/jeu.70011 (PMC12079164; doi:10.1111/jeu.70011)
Supplement: Supplementary file 8 — Table S4. Approximately unbiased (AU) test for alternative topologies and taxon placement in phylogenetic analyses. [file JEU-72-e70011-s004.docx]

**Table S4**. Approximately Unbiased (AU) Test for Alternative Topologies and Taxon Placement in Phylogenetic Analyses.

| **Tree** | **p-AU** |
| --- | --- |
| ***Hypothesis I*** | |
| Main Tree Fig. 5. ((IBL,(Pe,(PA,(E,(D,(A,K)))) | 0.4 |
| Alternative: ((IBL,(Pe,(PA,((E,D),(A,K))))) | 0.3 |
| ***Hypotheses II*** | |
| Main Tree Fig. 5. ((IBL,(Pe,(PA,(E,(D,(A,K)))) | 0.035* |
| Alternative: (((PA,(E,(D,(A,K)))),IBL), Pe) | 0.241 |
| Alternative: (((PA,(E,(D,(A,K)))),Pe), IBL) | 0.877 |

*p<0.05 is rejected.

Taxon and clade name key: ***IBL*** *- (P. invadens + P. branchiphila) + Neoparamoeba longipodia);* ***PA*** *- P. pemaquidensis + P. aestuarina;* ***Pe*** *- P. perurans;* ***E*** *- P. eilhardi;* ***D*** *- P. Dayton sp. n.;* ***A*** *- P. aparasomata;* ***K*** *- P. kareshi.*
